# *CmWRKY1* Enhances the Dehydration Tolerance of Chrysanthemum through the Regulation of ABA-Associated Genes

**DOI:** 10.1371/journal.pone.0150572

**Published:** 2016-03-03

**Authors:** Qingqing Fan, Aiping Song, Jiafu Jiang, Ting Zhang, Hainan Sun, Yinjie Wang, Sumei Chen, Fadi Chen

**Affiliations:** 1 College of Horticulture, Nanjing Agricultural University, Nanjing, 210095, China; 2 Jiangsu Province Engineering Lab for Modern Facility Agriculture Technology & Equipment, Nanjing, 210095, China; Key Laboratory of Horticultural Plant Biology (MOE), CHINA

## Abstract

WRKY transcription factors serve as antagonistic or synergistic regulators in a variety of abiotic stress responses in plants. Here, we show that CmWRKY1, a member of the group IIb WRKY family isolated from *Chrysanthemum morifolium*, exhibits no transcriptional activation in yeast cells. The subcellular localization examination showed that CmWRKY1 localizes to the nucleus *in vivo*. Furthermore, *CmWRKY1*-overexpressing transgenic lines exhibit enhanced dehydration tolerance in response to polyethylene glycol (PEG) treatment compared with wild-type plants. We further confirmed that the transgenic plants exhibit suppressed expression levels of genes negatively regulated by ABA, such as *PP2C*, *ABI1* and *ABI2*, and activated expression levels of genes positively regulated by ABA, such as *PYL2*, *SnRK2*.*2*, *ABF4*, *MYB2*, *RAB18*, and *DREB1A*. Taken together, our results indicate that *CmWRKY1* plays an important role in the response to drought in chrysanthemum through an ABA-mediated pathway.

## Introduction

Many WRKY transcription factors have been identified since the first WRKY gene, defined as *SPF1*, was cloned from sweet potato (*Ipomoea batatas*) [1]. The transcription factors of the WRKY family possess a WRKY domain consisting of a conserved WRKYGQK heptapeptide at the N terminus with a C_2_H_2_- or C_2_HC-type zinc-finger-motif structure at the C terminus [2]. It has been clearly established that the WRKY family can be classified into three groups based on construction criteria. The WRKY proteins in group I are composed of two WRKY domains and two C_2_H_2_ zinc fingers, whereas only one WRKY domain and one C_2_H_2_ zinc finger are present in the group II proteins, and a single C_2_HC zinc finger and one WRKY domain are found in the members of group III. Furthermore, the group II proteins are clearly divided into five subgroups (IIa, IIb, IIc, IId and IIe) [3]. Recent studies have focused on the role of multiple WRKY factors as players linked to abiotic stresses, including drought, salt, cold and heat stress. Furthermore, this research field is no longer limited to the model species Arabidopsis and is spreading to numerous other species [2,4]. For example, the regulation of *OsWRKY11* in rice (*Oryza sativa*) by the *HSP101* promoter resulted in increased heat and drought tolerance [5]. Similarly, the overexpression of *GmWRKY54* in soybean (*Glycine max*) plants leads to markedly higher drought tolerance [6]. In contrast, *CmWRKY17* in chrysanthemum enhances the sensitivity of transgenic chrysanthemum to salt stress [7].

The group II WRKY proteins have been reported to participate in plant processes such as senescence and germination and in the response to abiotic stresses such as drought, salt and cold [3]. The first WRKY protein to be recognized as a pivotal player in senescence was AtWRKY6, which is strongly induced during senescence [8]. The available evidence suggests that many group II WRKY proteins play a major role in drought stress. For instance, *LcWRKY5*, isolated from sheepgrass (*Leymus chinensis*), is a member of the group IIa WRKY proteins. The overexpression of *LcWRKY5* in Arabidopsis strongly enhances dehydration resistance by regulating the *DREB2A* pathway [9]. Similarly, *BdWRKY36* cloned from *Brachypodium distachyon* belongs to group IIe of the WRKY family, and its expression is upregulated following drought application. Additionally, the overexpression of *BdWRKY36* in transgenic tobacco (*Nicotiana tabacum*) increases resistance to drought by reducing the accumulation of reactive oxygen species (ROS) and activating the stress defense-related gene *NtLEA5*, the ABA biosynthesis-related gene *NtNCED1* and the regulatory gene *NtDREB3* [10]. Moreover, BhWRKY1 (homologous to AtWRKY60) is a member of the group II WRKY proteins and is stimulated by dehydration and ABA in a rapid and transient manner. *BhWRKY1* may interact with *BhGolS1* to act as a signal in an ABA-dependent pathway that contributes to enhanced dehydration tolerance in transgenic tobacco [11].

Many WRKY transcription factors have been investigated to better understand the breadth of their involvement in ABA-associated plant processes, such as seed dormancy and germination, as well as the ABA-mediated signaling network. ABA has emerged as a prominent regulator of drought tolerance in plants through influencing the movements of stomata [12]. It was previously reported that AtWRKY2 from Arabidopsis is likely to antagonistically regulate ABA-related seed germination and post-germination growth [13]. Previous reports indicate that AtWRKY40 from group II serves as a transcriptional repressor by acting as a negative regulator in the ABA signaling network [14]. Moreover, *CmWRKY15* from chrysanthemum facilitates *Alternaria tenuissima* infection by regulating the ABA signaling network [15]. Moreover, *CmWRKY48*-overexpressing chrysanthemum plants are able to inhibit the population growth of aphids [16]. A recent study found that *GhWRKY68*, which is characterized as a group IIc WRKY, can be obtained from cotton (*G*. *hirsutum L*.) and that the overexpression of this protein in *Nicotiana benthamiana* increases the plants’ sensitivity to drought and salt stresses by modulating ABA signaling [17].

Chrysanthemum (*Chrysanthemum morifolium*) is one of the leading ornamental cut flowers worldwide, and its production faces a variety of challenges under environmental stress conditions [18,19]. Drought stress is one of the most harmful types of stress because it retards chrysanthemum growth. Hence, it is essential to improve the tolerance of chrysanthemum to this type of stress in order to achieve sustainable production. Therefore, an investigation of chrysanthemum under drought stress conditions with the aim of elucidating the relevant mechanisms at the molecular level will help alleviate the effects of drought and have both theoretical and practical importance. *WRKY* genes serve as critical factors in plant drought tolerance [20]. *CmWRKY1*, which is cloned from chrysanthemum, is a characteristic gene belonging to the group IIb WRKY superfamily. *CmWRKY1* shares homology with AtWRKY6 of *A*. *thaliana*, which is down-regulated by exogenous ABA treatment but markedly induced by moisture stress [21]. In this study, we emphasize the mechanism through which *CmWRKY1* modulates the ABA-mediated pathway in chrysanthemum in response to drought.

## Materials and Methods

### Plants Materials and Growth Conditions

The chrysanthemum cultivar ‘Jinba’ was obtained from the Chrysanthemum Germplasm Resource Conservation Centre, Nanjing Agricultural University, China. We propagated uniform seedlings in pots using a 1:1 (v/v) mixture of soil and vermiculite and cultivated the plants in a greenhouse under day and night temperatures of 25 and 18°C, respectively, a 14-h light/10-h dark photoperiod, a light intensity of 50 μmol m^-2^s^-1^ and a relative humidity of 70%.

### Phylogenetic Analysis of CmWRKY1 Orthologs

The CmWRKY1 amino acid sequence was aligned with the sequences of its homologs using DNAman 5.2.2 software and BLAST online software (http://www.ncbi.nlm.gov/blast). The sequences of genes orthologous to *CmWRKY1* were acquired from the NCBI database (http://www.ncbi.nlm.nih.gov), and we then aligned the sequences of *WRKY* genes from different species using ClustalW software [22]. To obtain better classifications of the multiple branches, phylogenetic trees including 22 representative homologs from the CmWRKY1 sequence analysis were generated with the MEGA6 program by employing the neighbor-joining method with 1000 bootstrap replicates [23].

### Analysis of CmWRKY1 Transcriptional Activity

We employed a yeast assay system to test the transcriptional activation of CmWRKY1. To amplify the ORF of *CmWRKY1* without the termination codon, the Phusion^®^ High-Fidelity PCR Kit (New England Biolabs, Ipswich, MA, USA) was used with the primer pair *CmWRKY1*-GATE-SAL-F/*CmWRKY1*-GATE-NOT-R (S1 Table). We performed *Sal* I/*Not* I double digestion and ligation to import the PCR products into the pENTR^™^1A vector (Invitrogen, Carlsbad, CA, USA). With the aid of the LR Clonase^™^ II enzyme mix (Invitrogen), pENTR^™^1A-*CmWRKY1* and pDEST-GBKT7 were recombined into pDEST-GBKT7-*CmWRKY1*. The pDEST-GBKT7-*CmWRKY1* construct, pCL1 (positive control) and pGBKT7 (negative control) were then inserted into the *Saccharomyces cerevisiae* Y2HGold strain (Clontech) following the manufacturer’s recommended procedure. The selection of transformants was performed for either pGBKT7-*CmWRKY1* or pGBKT7 using synthetic dropout (SD)/-Trp medium, whereas pCL1 was selected on (SD)/-Leu medium. Y2H cells containing pCL1, which can grow on SD/-His-Ade medium, were used as a positive control, whereas Y2H cells carrying pGBKT7, which were used as a negative control, cannot grow on this medium.

We utilized the CmWRKY1 luminescence assay to determine its transcriptional activity. The CmWRKY1 ORF was amplified by PCR using the primer set CmWRKY1-GATE-F/R (S1 Table) containing the BamH I and Not I sites to obtain the 35S::GAL4DBD-CmWRKY1 fusion component. The amplified DNA fragment was then inserted into the pENTRTM1A dual selection vector (Invitrogen) to acquire pENTRTM1A-CmWRKY1 after sequencing. We subjected the plasmid to the 35S::GAL4DBD vector to generate the structure of 35S::GAL4DBD-CmWRKY1 employing the LR reaction (Invitrogen). Protoplasts of Arabidopsis were obtained and transfected according to the protocol previously described by Yoo et al. [24]. During the transfection, we transfected 7.5 μg of 35S::GAL4DBD-AtARF5, 35S::GAL4DBD or 35S::GAL4DBD-CmWRKY1, with an additional 7.5 μg of 5×GAL4-LUC as the reporter plasmid and a reporter gene for luciferase driven by five copies of GAL4-binding elements. The luciferase activities were assessed as previously described [25]. Three independent experiments were performed.

### Subcellular Localization of CmWRKY1

A transient assay was performed to ascertain the subcellular localization of CmWRKY1 by transforming the construct into onion epidermal cells. The LR Clonase^™^ II enzyme mix (Invitrogen) was used to generate a green fluorescent protein (GFP)-*CmWRKY1* fusion to obtain the plasmid construct pENTR^™^1A-*CmWRKY1* in pMDC43. We then transiently transformed the *p35S*::*GFP*-*CmWRKY1* constructs and the empty pMDC43 vector as a marker into onion epidermal cells. The expression of GFP was detected via confocal laser microscopy.

### Transformation Procedure to Obtain *CmWRKY1*-Overexpressing Chrysanthemum

To further analyze the function of *CmWRKY1*, *CmWRKY1*-overexpressing chrysanthemum transformants were obtained. We transformed the *35S*::*CmWRKY1* plasmid into the *Agrobacterium tumefaciens* construct EHA105 using the freeze-thaw transformation method.

The procedure used for the transformation of chrysanthemum was described previously [7,26]. We initially obtained explants from leaf discs (5 mm in diameter) collected from sterile ‘Jinba’ plants cultured *in vitro* and then obtained the target transformants by cultivating them on medium supplemented with 8 mg L^-1^ hygromycin. After regeneration, we extracted RNA from the putative transgenic and wild-type plants using the RNAiso reagent (TaKaRa). DNA was eliminated with RNase-free DNase I (TaKaRa), and reverse transcription was performed with M-MLV reverse transcriptase (TaKaRa). The relative expression of *CmWRKY1* was determined through quantitative real-time PCR (qRT-PCR) analysis using the SYBR^®^ Green reaction kit (TaKaRa) with the primer pair *CmWRKY1*-DL-F/R (S1 Table). The reference gene *CmEF1α* was amplified using the primer pair CmEF1α-F/R (S1 Table). We utilized a Mastercycler ep realplex device (Eppendorf, Hamburg, Germany) to run the qPCR assays. The transcription data (with three biological replicates for each sample) were calculated using the 2^−ΔΔCt^ method [27].

### Drought Treatment of *CmWRKY1*-Overexpressing Transgenic Chrysanthemum and Wild-Type Plants

Groups of 50 cuttings were acquired from both the transgenic chrysanthemum plants (W1 plants) and the wild-type ‘Jinba’ (wild-type plants) to determine their drought tolerance, and 10 cuttings from each group were used as a control. The cuttings were maintained at 22 ± 3°C under a 14-h light/10-h dark photoperiod in aerated water for 14 d. When the cuttings had grown to the six-to-eight-leaf stage, the treated plants were exposed to drought in plastic cups containing 20% PEG6000, whereas the control cuttings were treated with water. After treatment, the plants were maintained in a greenhouse (day/night temperatures of 25/18°C, a light/dark photoperiod of 14/10 h, a light intensity of 50 μmol m^-2^s^-1^ and a relative humidity of 70%). At 0 h, 1 h, 8 h, 12 h, 24 h and 36 h after exposure to PEG, the third true leaf from three seedlings each of the W1-1, W1-2 and WT plants under each treatment was collected. Each experiment included three biological replicates. Samples collected at defined time points for each treatment were pooled for RNA extraction. After treatment for 48 h, the roots were washed with water, and the plants were returned to fresh water and allowed to recover for one additional week, after which time the survival rate was calculated [19,28].

Each group of 10 cuttings from the W1 and wild-type plants was cultivated in the aperture disk to determine the plants’ drought tolerance. When the cuttings had grown to the six-to-eight-leaf stage, they were not watered for ten days, after which time we began to water them again and allowed the cuttings to recover for one additional week.

### Measurement of Relative Water Content (RWC)

Uniform cuttings were propagated in a pot using a 1:1 (v/v) mixture of soil and vermiculite and cultivated in a greenhouse (day/night temperatures of 25/18°C, a 14-h light/10-h dark photoperiod, a light intensity of 50 μmol m^-2^s^-1^ and a relative humidity of 70%). Each treatment comprised 20 seedlings of each of the transgenic chrysanthemum and wild-type plants. After treatment, the plants were maintained in a greenhouse (day/night temperatures of 25/18°C, a light/dark photoperiod of 14/10 h, a light intensity of 50 μmol m^-2^s^-1^ and a relative humidity of 70%). At 0 h, 4 h, 12 h and 36 h after exposure to PEG, we removed the third true leaf from three seedlings each of the W1-1, W1-2 and WT plants under each treatment. Each experiment included three biological replicates. Samples collected at defined time points from each treatment were used for measuring the relative water content (RWC). To obtain this measurement, the leaves were detached immediately and weighed to obtain the fresh weight (FW). The leaves were then incubated in distilled water for 24 h at room temperature under normal light, and the turgid weight (TW) was then recorded. The samples were then transferred into an oven at 80°C for 48 h, and the dry weights (DW) were recorded. The RWC was calculated according to Sun [10]: RWC (%) = [(FW—DW)/(TW- DW)] ×100%.

## Results

### Phylogenetic Analysis of CmWRKY1-Homologous Sequences

**Fig 1:**
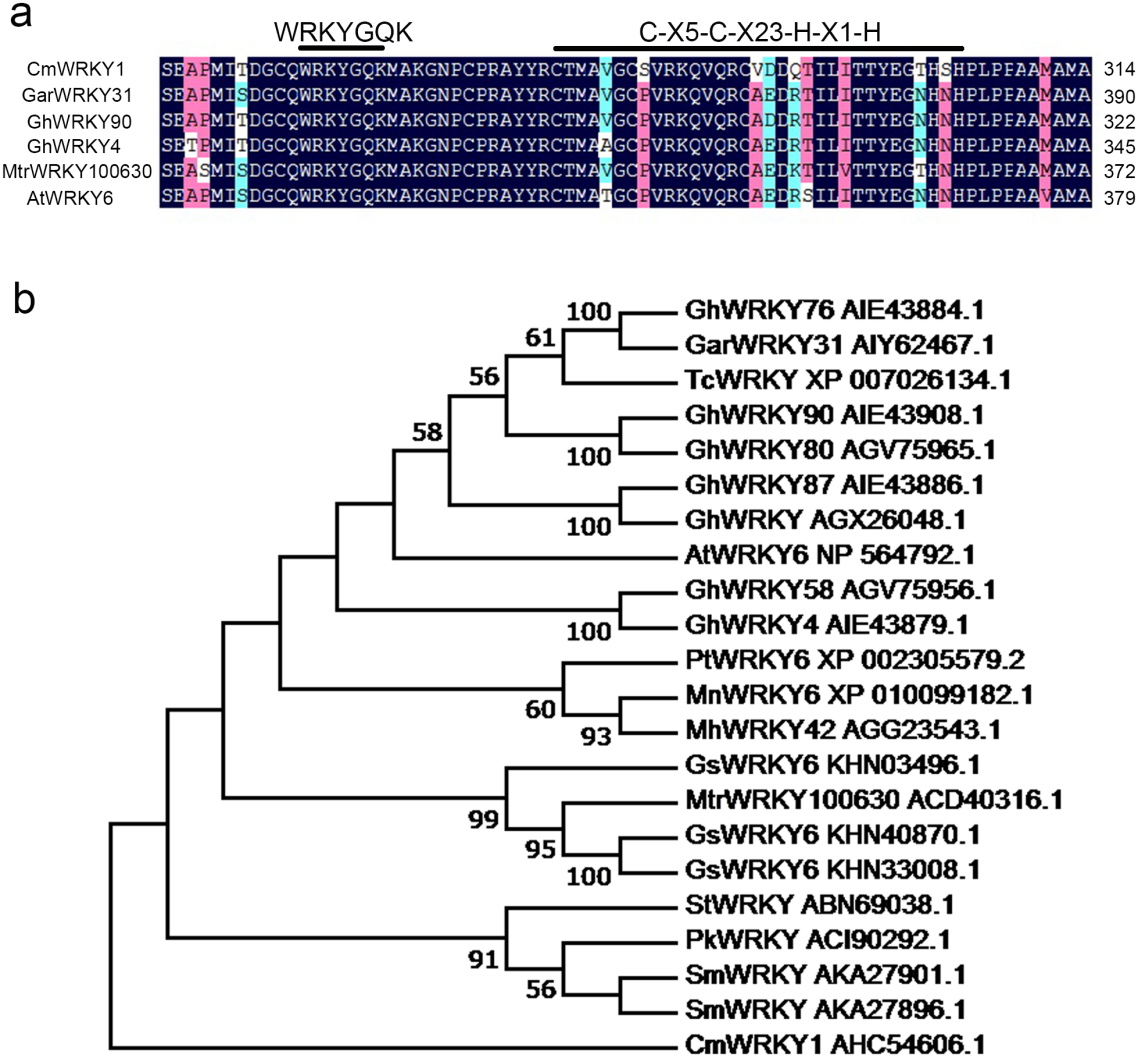
Deduced peptide sequences of CmWRKY1 and other WRKY proteins. **a** Alignment of the putative amino acid sequence of CmWRKY1 with the amino acid sequences of homologous proteins. Features of the sequence include a WRKY domain (WRKYGQK) and a C_2_H_2_ zinc finger domain (both highlighted with lines above the alignment). The accession numbers for the sequences are listed below. GarWRKY31 (AIY62467.1), GhWRKY90 (AIE43908.1), GhWRKY4 (AIE43879.1), MtrWRKY100630 (ACD40316.1), AtWRKY6 (NP_564792.1). **b** Phylogenetic tree showing homologs of CmWRKY1 and WRKY proteins from other species. ClustalW was used to align the amino acid sequences, and the neighbor-joining method was used to construct the phylogenetic tree with MEGA 6.0. The accession numbers for the sequences are listed below. CmWRKY1 (AHC54606.1), PkWRKY (ACI90292.1), TcWRKY (XP_007026134.1), GhWRKY76 (AIE43884.1), GarWRKY31 (AIY62467.1), GhWRKY90 (AIE43908.1), GhWRKY58 (AGV75956.1), SmWRKY (AKA27901.1), MnWRKY6 (XP_010099182.1), PtWRKY6 (XP_002305579.2), GhWRKY4 (AIE43879.1), GsWRKY6 (KHN40870.1), GhWRKY87 (AIE43886.1), MhWRKY42 (AGG23543.1), MtrWRKY100630 (ACD40316.1), GhWRKY (AGX26048.1), AtWRKY6 (NP_564792.1), GsWRKY6 (KHN33008.1), StWRKY (ABN69038.1), GsWRKY6 (KHN03496.1), SmWRKY (AKA27896.1), GhWRKY80 (AGV75965.1).

The alignment of CmWRKY1 with GarWRKY31 [29], GhWRKY90 [30], GhWRKY4 [30], MtrWRKY100630 [31] and AtWRKY6 [32] showed that CmWRKY1 contains one WRKY domain (WRKYGQK) and one C_2_H_2_ zinc-finger motif (C-X5-C-X23-H-X1-H) (Fig 1a). A phylogenetic analysis showed that CmWRKY1 is most closely related to SmWRKY from *Solanum tuberosum* and exhibits higher similarity to AtWRKY6 in Arabidopsis. Moreover, CmWRKY1 displays high similarity to GhWRKY4 and GhWRKY90 from *Gossypium hirsutum*, GarWRKY31 from *Gossypium aridum* and MtrWRKY100630 from *Medicago truncatula* (Fig 1b).

### Subcellular Localization of CmWRKY1

**Fig 2:**
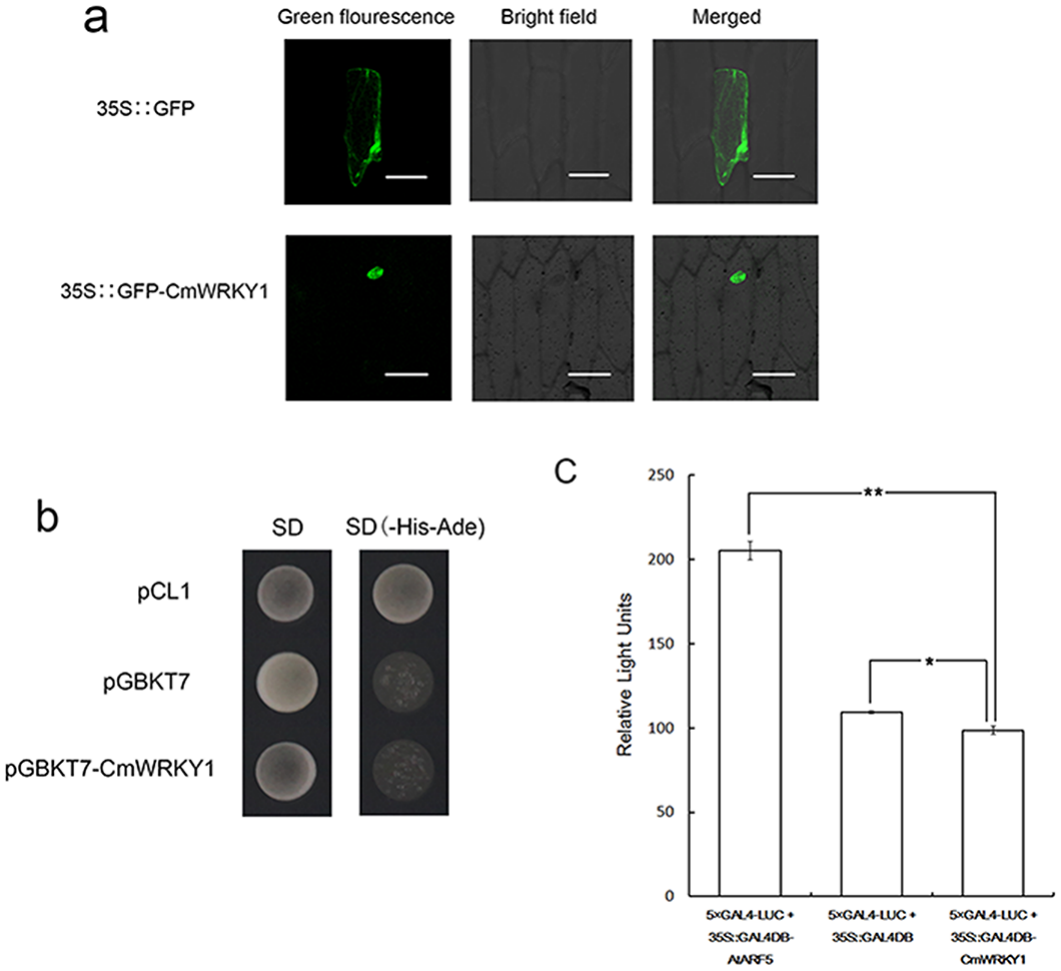
Analysis of the subcellular localization and transactivation of CmWRKY1. The transactivation analysis was performed using a yeast assay system. **a** Subcellular localization of CmWRKY1. **b** Analysis of the transcriptional activity of CmWRKY1 in a yeast assay system. The Y2H cells containing pCL1, which served as a positive control, can grow on SD/-His-Ade medium, whereas the Y2H cells containing pGBKT7, which served as a negative control, cannot grow on this medium. **c** Relative luciferase activities in *Arabidopsis* mesophyll protoplasts after transfection with 35S::GAL4DBD-CmWRKY1.

We transformed the *35S*::*GFP*-*CmWRKY1* construct and a positive vector harboring only *35S*::*GFP* into onion epidermal cells via particle bombardment. Onion epidermal cells containing *35S*::*GFP* presented GFP fluorescence throughout the cells (Fig 2a). In contrast, GFP fluorescence was observed only in the nucleus of the onion epidermal cells harboring the GFP-CmWRKY1 fusion protein (Fig 2a). These findings indicate that *CmWRKY1* localizes to the nucleus *in vivo*.

### CmWRKY1 Transactivation Assay

The transcriptional activation of CmWRKY1 was assessed utilizing a yeast one-hybrid system. Yeast harboring the negative control pGBKT7 and the pGBKT7-*CmWRKY1* construct were unable to grow on SD/-His-Ade medium due to yeast cell shelter, but the positive control pCL1 grew well (Fig 2b). These results demonstrate that *CmWRKY1* exhibits no transcriptional activation in yeast cells.

Because we found that CmWRKY1 shows no transcriptional activation in yeast cells, to further understand the transactivation function of CmWRKY1, we transfected the plasmid encoding CmWRKY1 into Arabidopsis protoplasts in combination with a reporter plasmid. The results (Fig 2c) show that the relative LUC units (RLUs) of 35S::GAL4DBD-AtARF5 were significantly higher than those of 35S::GAL4DBD-CmWRKY1 in Arabidopsis protoplasts (*P*<0.01), whereas the RLUs of 35S::GAL4DBD-CmWRKY1 were lower than those of 35S::GAL4DBD (*P*<0.05) (Fig 2c), indicating that CmWRKY1 serves as a repressor of transcription.

### *CmWRKY1* Overexpression Enhances the Dehydration Tolerance of Chrysanthemum

**Fig 3:**
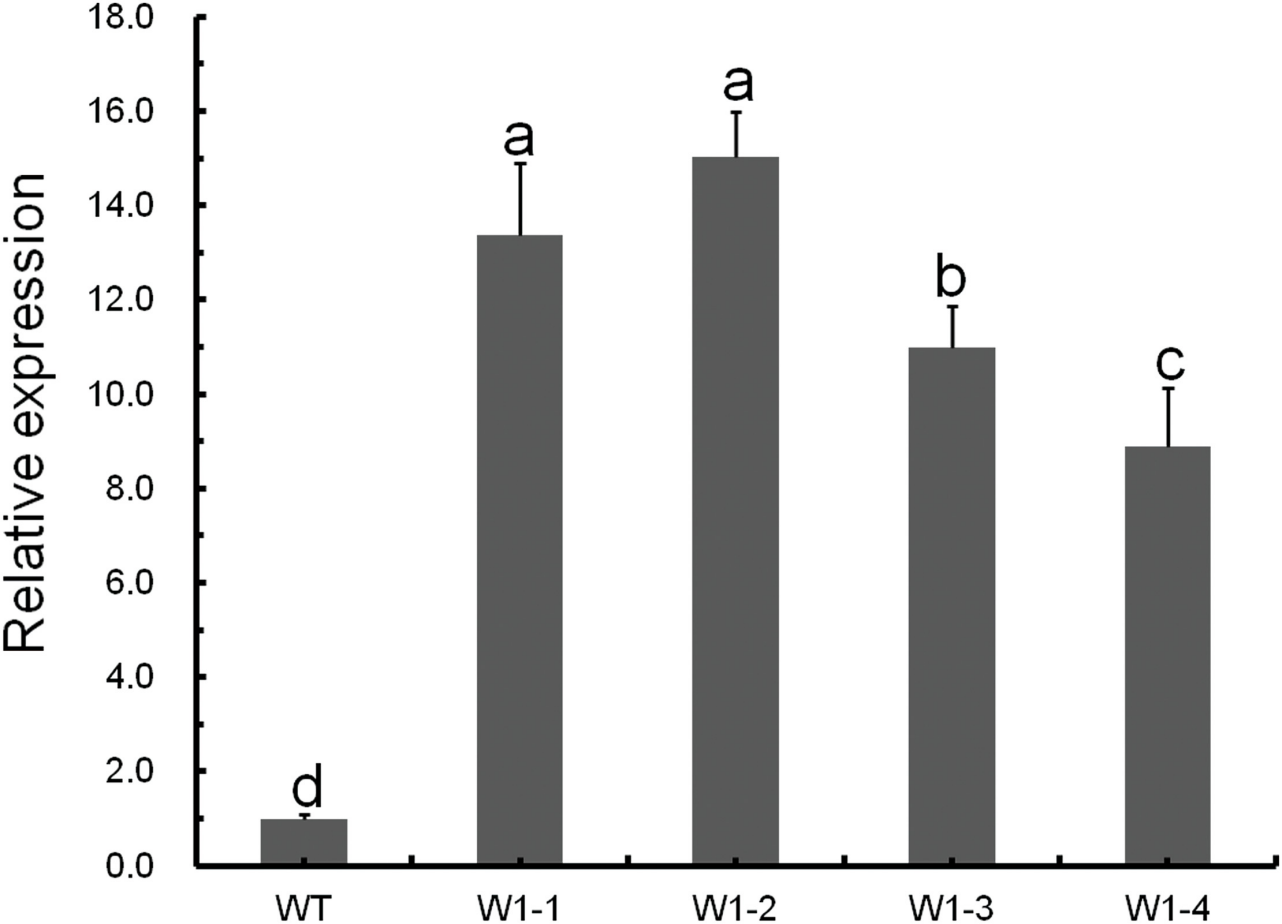
Relative expression levels of the *CmWRKY1* gene in transgenic plants. Expression of the *CmWRKY1* transcript in wild-type ‘Jinba’ and transgenic chrysanthemum lines overexpressing *CmWRKY1*.

**Fig 4:**
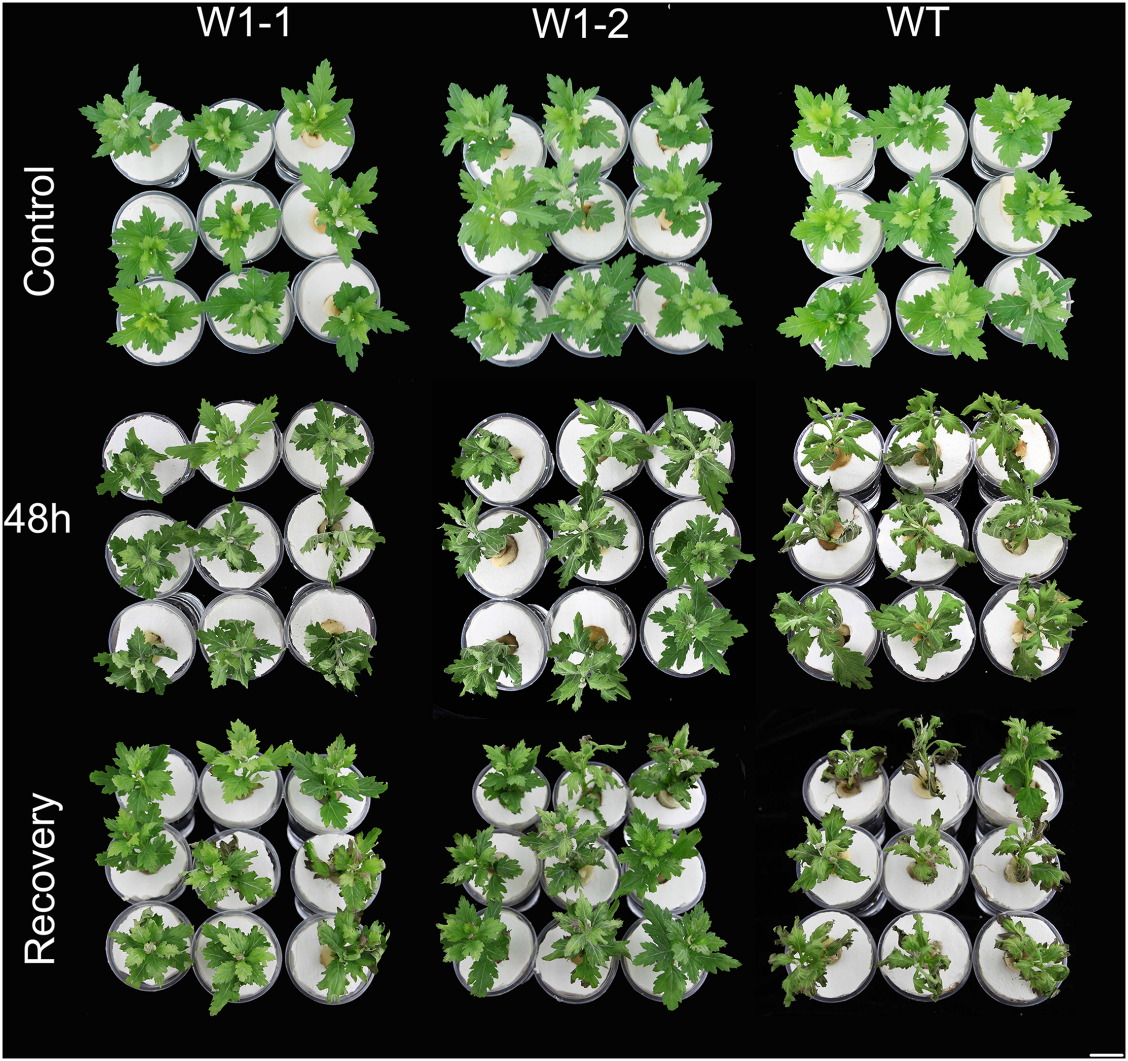
Phenotypic differences between transgenic chrysanthemum lines overexpressing *CmWRKY1* and wild-type ‘Jinba’ under PEG 6000 treatment. When the cuttings had grown to the six-to-eight-leaf stage, the treated samples were exposed to drought in plastic cups containing 20% PEG6000, whereas the control cuttings were treated with water. After treatment for 48 h, the roots were washed with water, and the plants were returned to fresh water and allowed to recover for one additional week.

**Fig 5:**
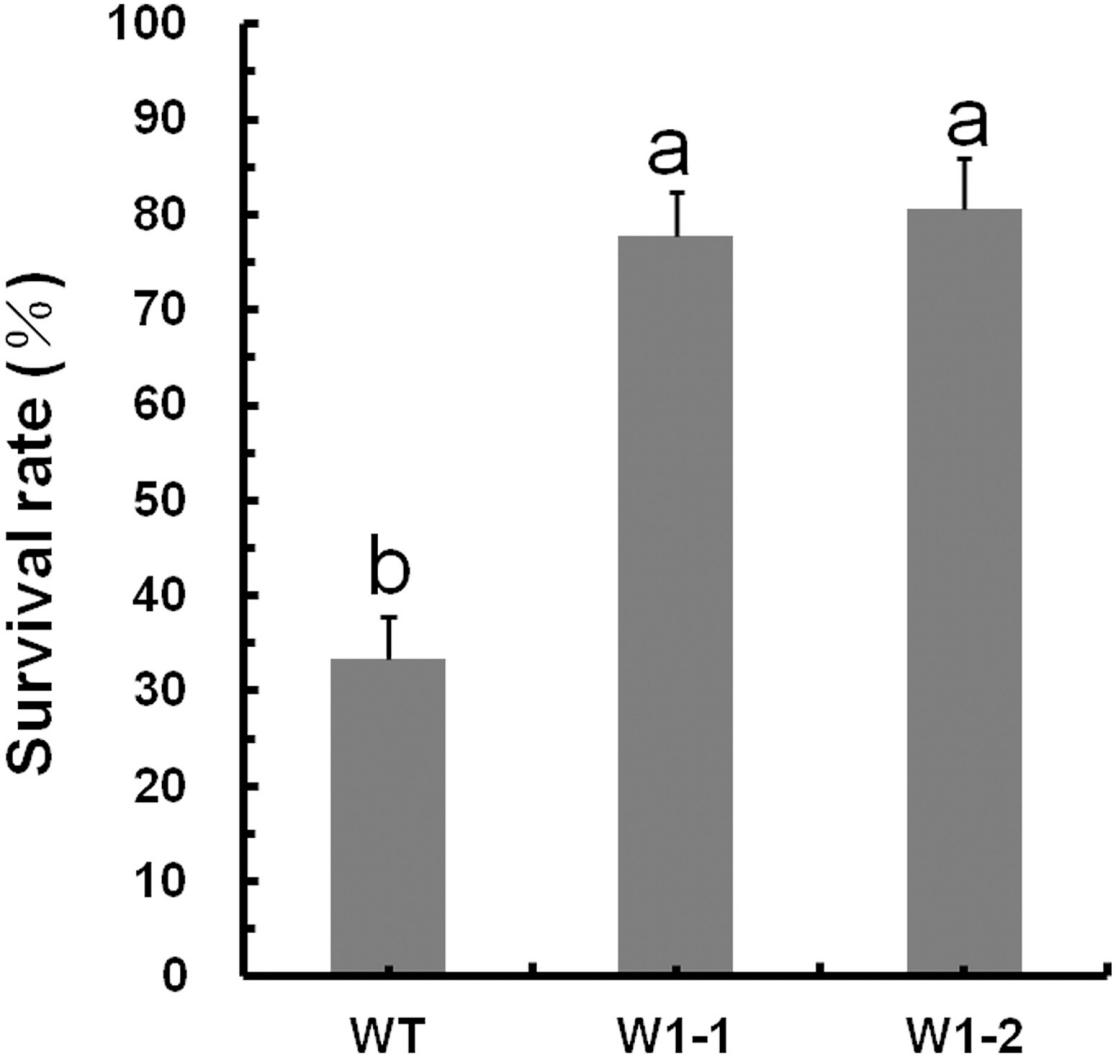
Survival rates of wild-type ‘Jinba’ and transgenic chrysanthemum plants under PEG 6000 treatment.

**Fig 6:**
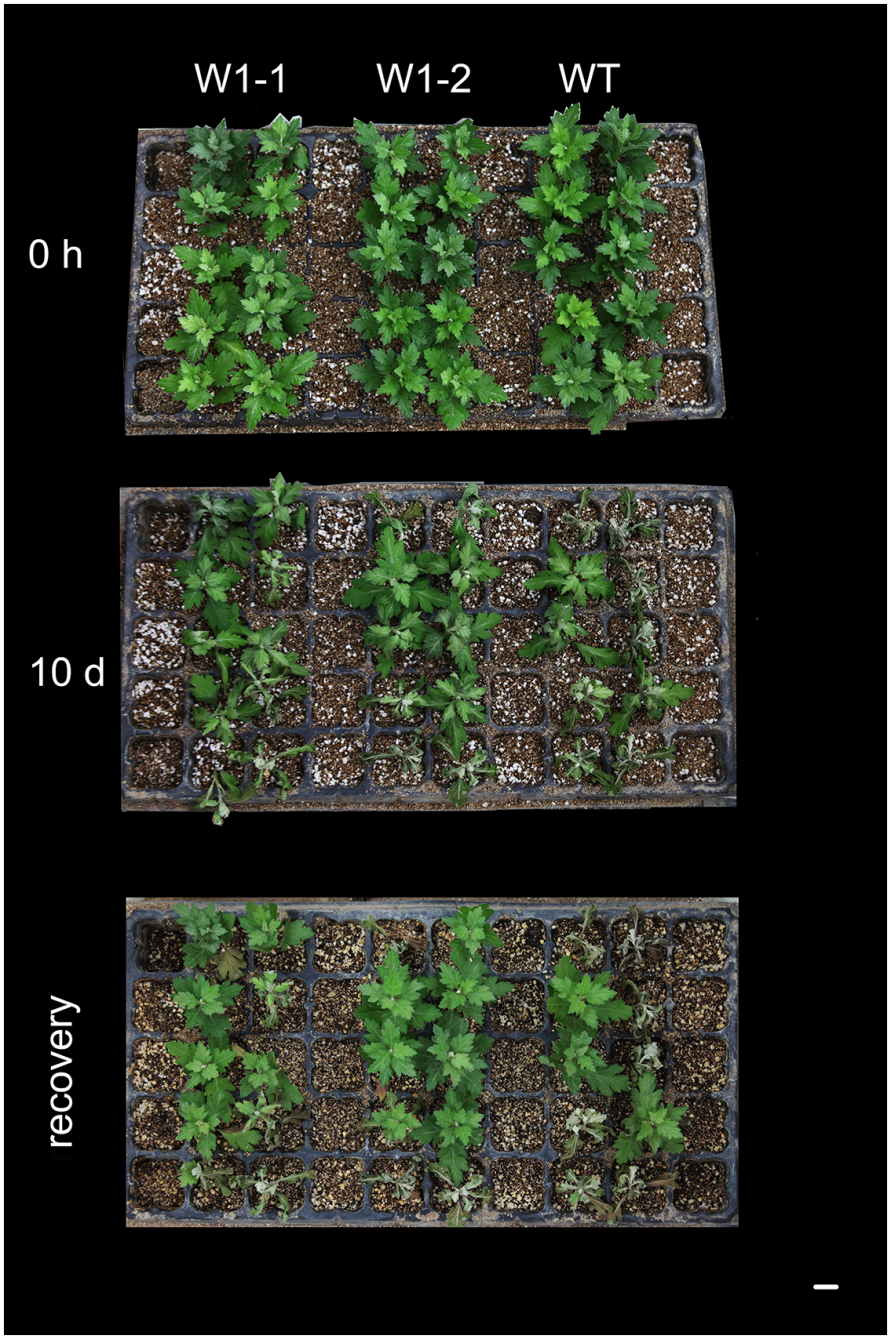
Phenotypic differences between transgenic chrysanthemum lines overexpressing *CmWRKY1* and wild-type ‘Jinba’ plants under drought treatment. Each group of 10 cuttings from W1 and wild-type plants was cultivated in an aperture disk to assessment of their drought tolerance. When the cuttings had grown to the six-to-eight-leaf stage, watering was halted for ten days, and after this time, the cuttings were watered again and allowed to recover for one additional week.

In this study, we employed *CmWRKY1*-overexpressing transgenic chrysanthemum lines, and the transcript levels of *CmWRKY1* in these plants were determined through qPCR. In the *CmWRKY1*-overexpressing plants, the *CmWRKY1* (W1-1 and W1-2) transcript levels were significantly higher than those observed in wild-type plants (Fig 3). Two lines showing relatively higher *CmWRKY1* expression, namely W1-1 and W1-2, were selected for PEG treatment. After PEG treatment for 48 h, the plants were severely wilted with withered leaves. The degree of leaf withering in the wild-type plants was markedly more severe than that observed in W1-1 and W1-2 plants (Fig 4). After the recovery period, the survival rates of the wild type, W1-1 and W1-2 plants were 13.3%, 51.7% and 56.7%, respectively (Fig 5). After halting watering for ten days, the cuttings in the aperture disk were severely wilted and presented withered leaves. The degree of leaf withering in the W1-1 and W1-2 plants was less severe compared with that observed in the wild-type plants (Fig 6). Considering these results together, significant differences in dehydration were evident between the *CmWRKY1*-overexpressing and wild-type plants, with the *CmWRKY1*-overexpressing lines showing increased water loss. Our results indicate that *CmWRKY1* overexpression enhances the tolerance of chrysanthemum to dehydration stress.

### Analysis of Relative Water Content (RWC) in Transgenic Chrysanthemum and Wildtype ‘Jinba’ Plants

**Fig 7:**
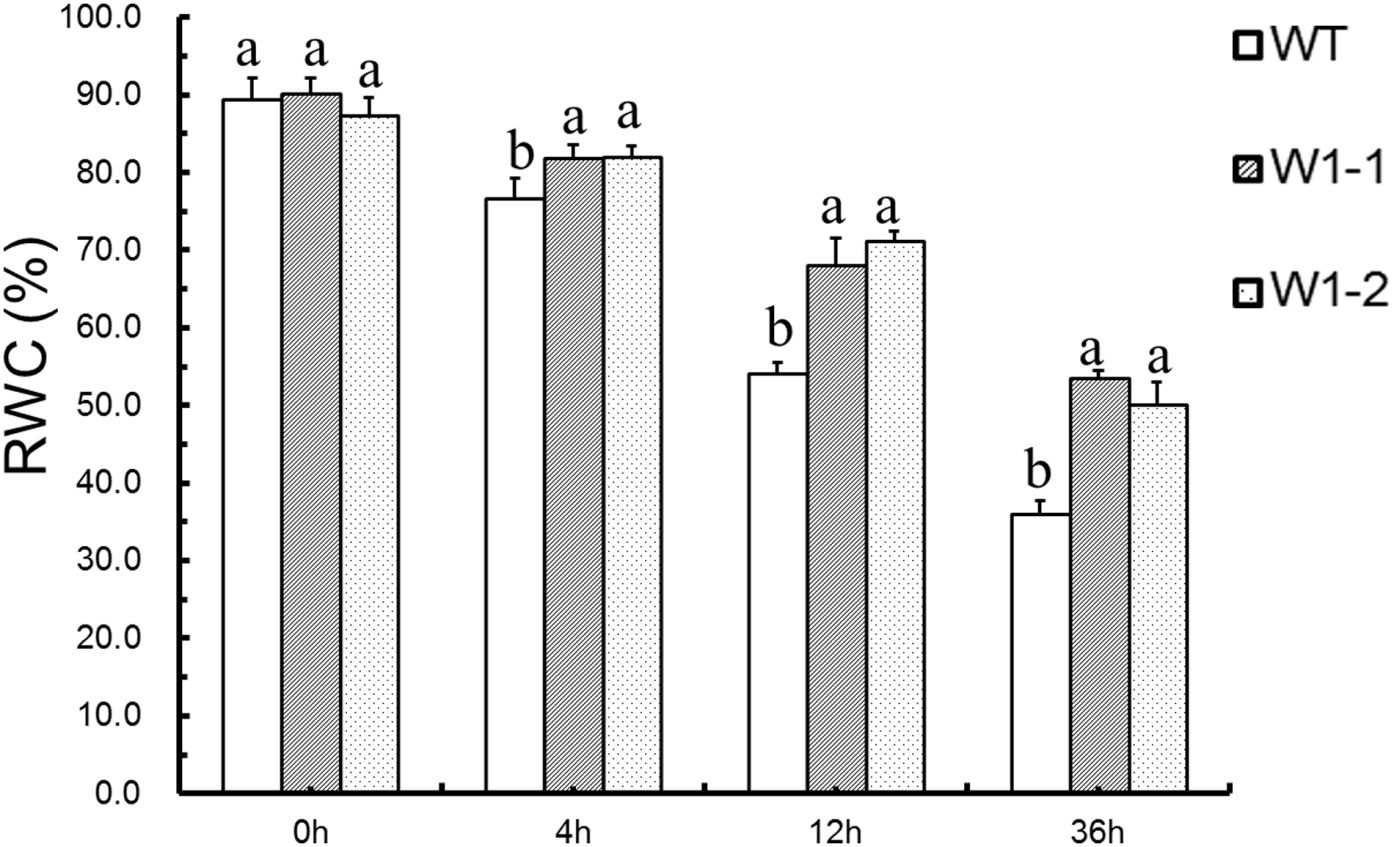
RWC in transgenic chrysanthemum and wild type ‘Jinba’ chrysanthemum plants. The RWCs in WT, W1-1 and W1-2 plants were detected after 0 h, 4 h, 12 h and 36 h of exposure to PEG.

The RWCs in WT, W1-1 and W1-2 plants at 0 h, 4 h, 12 h and 36 h after exposure to PEG were determined. The results show that the RWCs presented only slight differences between the transgenic and WT plants at 0 h, whereas the transgenic plants exhibited higher RWCs than the WT plants after treatment with PEG for 4 h, 12 h and 36 h (Fig 7).

### Putative Model of the *CmWRKY1* Regulation of ABA-Responsive Genes in Response to Dehydration Treatment

**Fig 8:**
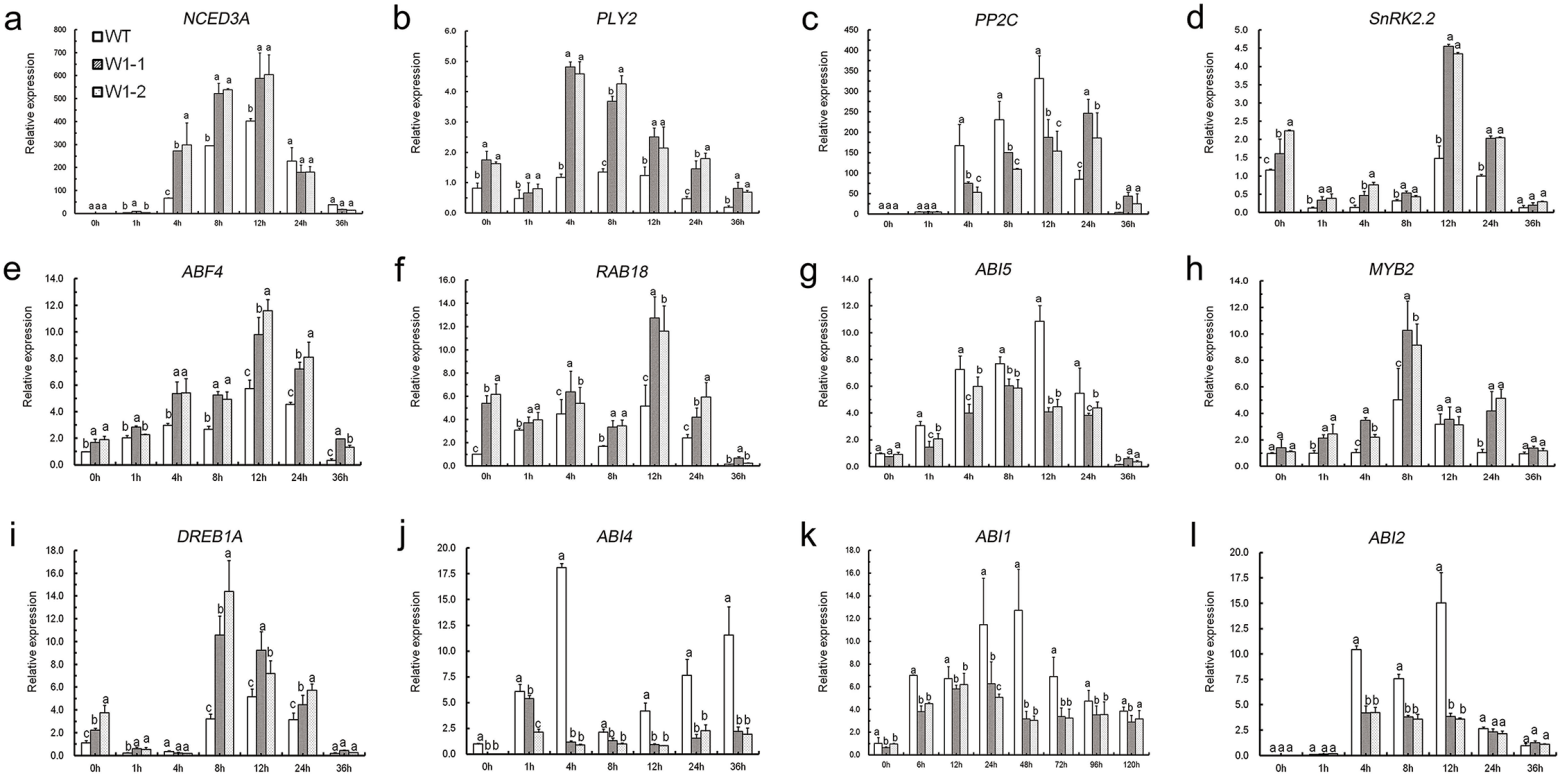
Expression of ABA-related genes in wild-type and *CmWRKY1* transgenic lines (W1-1 and W1-2).

To ascertain the regulatory mechanisms of *CmWRKY1* in response to dehydration stress, the expression levels of a group of ABA-associated genes, including *NCED3A*, *PP2C*, *PYL2*, *SnRK2*.*2*, *ABF4*, *RAB18*, *ABI5*, *MYB2*, *DREB1A*, *ABI4*, *ABI1* and *ABI2*, were compared between transgenic and wild-type plants (Fig 8). Under non-stress conditions, the expression of most ABA-responsive genes did not show considerable differences between the transgenic lines and wild-type plants. After dehydration stress, all of the examined ABA-associated genes were robustly activated. In contrast, the transcript levels of some negatively ABA-responsive genes (*PP2C*, *ABI4*, *ABI5*, *ABI1* and *ABI2*) were markedly reduced to varying degrees in *CmWRKY1*-overexpressing lines in comparison with the wild-type plants. In contrast, the expression levels of the positively ABA-regulated genes *NCED3A*, *PYL2*, *SnRK2*.*2*, *ABF4*, *MYB2*, *RAB18* and *DREB1A* were upregulated in the transgenic lines (Fig 8).

## Discussion

### Analysis of CmWRKY1 Homologues

A previous study revealed that AtWRKY6 can function as both a positive and a negative regulator of transcription [32]. An overwhelming amount of evidence has been obtained in further investigations of the breadth of the role of AtWRKY6 in the responses to diverse stresses. It was recently demonstrated that AtWRKY6 represses arsenate absorption through modulating arsenate transporter gene expression in Arabidopsis [33]. In tobacco, the *WRKY6* promoter has been used to form the *WRKY6*:*CKX1* construct. The *CKX1* gene encodes cytokinin oxidase/dehydrogenase and has been reported to confer tolerance to drought and salinity. Further research has shown that *WRKY6*:*CKX1* plants exhibit enhanced drought and heat tolerance due to a transient elevation of stomatal conductance, which is related to the facilitation of ABA catabolism [34]. Two transcription factors homologous to CmWRKY1 (GhWRKY4 and GhWRKY90 from *Gossypium hirsutum*), which are assigned to clades of group IIb in the WRKY family, have been shown to be significantly induced in leaves following drought stress [30]. It has also been suggested that *GhWRKY* genes are involved in the response to ABA and drought in cotton [30].

### Analysis of Relative Water Content (RWC) in Transgenic Chrysanthemum and Wildtype ‘Jinba’ Plants

Drought stress results in a retardation of plant growth through a cell physiology pathway. The RWC of plants can reflect their water retention capacity, and drought and dehydration stress result in reductions in the RWC, whereas plants with tolerance to these stresses exhibit increased RWCs [19]. The results show that the RWCs presented only slight differences between the transgenic and WT plants at 0 h, whereas the transgenic plants had higher RWCs than the WT plants after treatment with PEG for 4 h, 12 h and 36 h (Fig 7). These data suggest that the RWC decreases in response to dehydration stress and that the *CmWRKY1* gene may enhance the dehydration tolerance of transgenic chrysanthemum plants by inhibiting water loss.

### Overexpression of *CmWRKY1* in Chrysanthemum Facilitates Dehydration Tolerance

**Fig 9:**
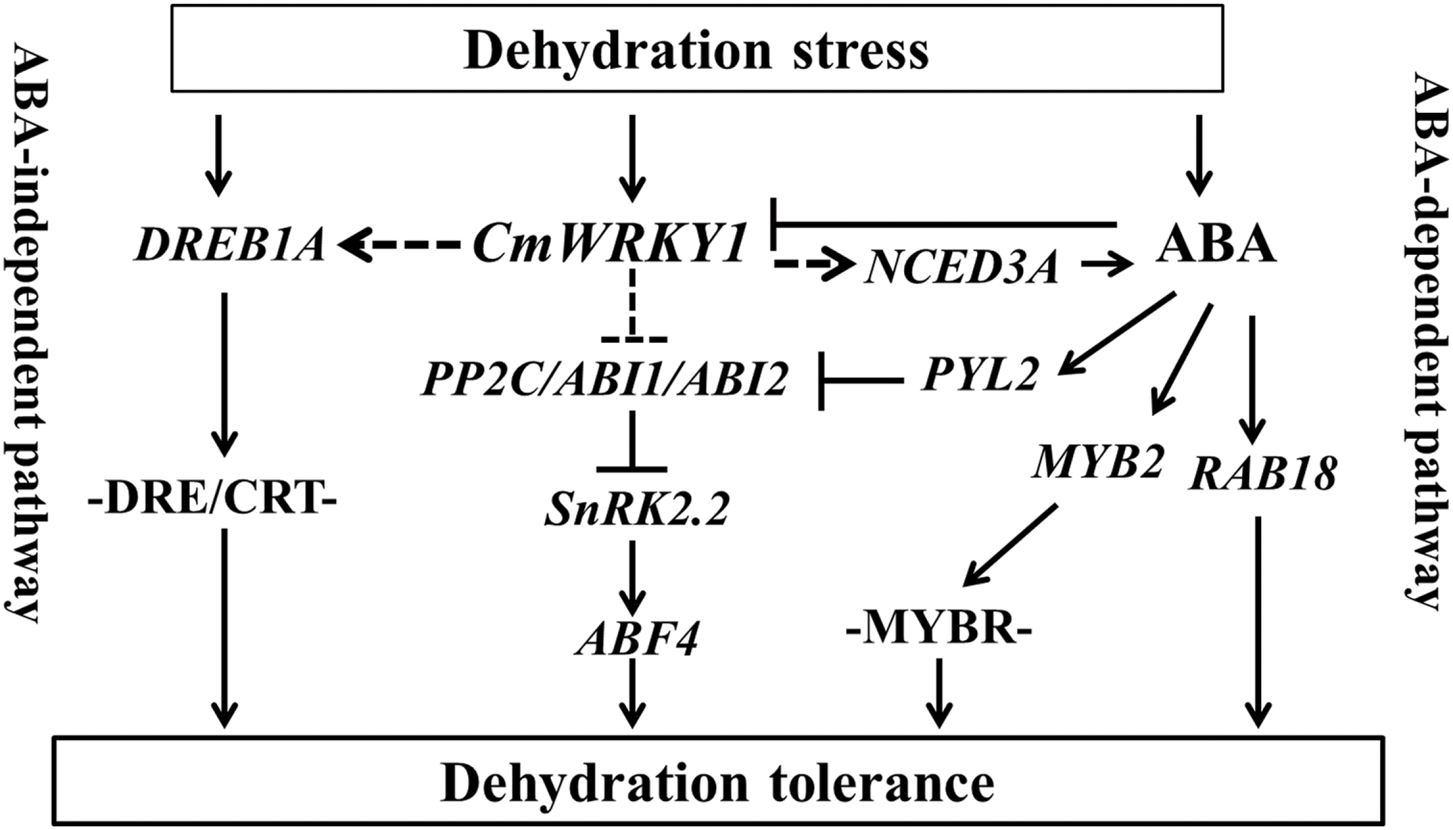
Model describing the interaction between *CmWRKY1* and ABA-associated genes in response to dehydration stress. A straight line means that the pathway has been reported, whereas a dotted line indicates a pathway identified in the present study.

Our findings suggest that *CmWRKY1* may facilitate dehydration tolerance by suppressing genes that respond negatively to ABA, such as *PP2C*, *ABI1* and *ABI2*, or by inducing genes that respond positively to ABA, such as *NCED3A*, *PYL2*, *SnRK2*.*2*, *ABF4*, *MYB2*, RAB18 and *DREB1A*. *CmWRKY1* can be down-regulated by exogenous ABA treatment but is markedly induced by dehydration stress [21], which supports a role for ABA in the inhibition of *CmWRKY1* expression and a role for dehydration stress in the stimulation of *CmWRKY1* expression (Fig 9).

### Suppression of PP2C, ABI1 and ABI2 by CmWRKY1

The type 2C protein phosphatases (PP2Cs), including PP2C, ABI1 and ABI2, have emerged as negative players in ABA-associated pathways that can dephosphorylate and inactivate SnRK2s in the absence of ABA [35]. *ABI1* (abscisic acid insensitive 1) and *ABI2* (abscisic acid insensitive 2), which are associated with PP2Cs, are negative regulators of the ABA signaling network. A previous study showed that the overexpression of *AtMYB20* improves salt tolerance and represses the expression of *ABI1*, *ABI2* and *AtPP2CA* [36]. Our results demonstrate that both *ABI1*, *ABI2* and *PP2C* are induced in wild-type plants and markedly repressed in W1-1 and W1-2 plants following water loss (Fig 8c, 8k and 8l). Hence, *CmWRKY1* may suppress the expression of PP2Cs to alleviate water deficit stress.

Recently, it was clearly demonstrated that SnRK2s interact with PP2Cs, leading to activation of the ABA signaling pathway [37]. It was previously shown that PP2Cs bind to SnRK2s to antagonistically regulate ABA-induced stomatal closure through inactivation of the slow-anion channel (SLAC1), which has been designated as an essential player in stomatal closure in guard cells [38,39].

It has been shown that among the SnRK2.2/SnRK2.3/SnRK2.6 clades of the subclass β SnRK2 family, SnRK2.2 and SnRK2.3 respond to ABA by activating ABA-related stress genes, such as abscisic acid-insensitive 5 (*ABI5*) and *ABF4* [40].

*ABI5* belongs to the bZIP superfamily and plays roles in ABA-dependent germination and post-germination growth [41,42]. In addition, *ABI5* has been found to act as a positive player in the ABA signaling network via *SnRK2*.*2* phosphorylation. *ABI5* is clearly induced after water loss. Interestingly, our findings show that *ABI5* was less activated in the overexpressing lines than in the wild-type ‘Jinba’ plants (Fig 8g). As a *SnRK2*.*2*-driven gene, the expression of *ABI5* should have been upregulated consistently with that of *SnRK2*.*2* in the overexpressing lines. The crosstalk between *CmWRKY1* and ABA-mediated signaling pathways is complex in plants under dehydration stress, and whether *CmWRKY1* directly inhibits *ABI5* remains a mystery.

ABF4, similar to ABRE2 (ABA responsive element), is an ABA-responsive element-binding factor. The AREB/ABFs belong to the group-A subfamily of leucine zipper (bZIP) transcription factors that contain one C-terminal conserved domain and three N-terminal domains [43]. A previous study showed that AREB2/ABF4 is significantly activated by water deficits, high salinity and exogenous ABA application [44]. Furthermore, the heterologous overexpression of *ZmbZIP72* from maize (*Zea mays*) enhances the drought tolerance of transgenic *Arabidopsis* plants and increases the expression of the ABA-inducing gene *RAB18* [45]. SnRK2s are reported to be involved in the phosphorylation and activation of bZIPs, including *ABFs* and *ABI5* [45,46].

The DREBs (dehydration-responsive element-binding factors) are members of the AP2/ERF (Apetala 2/ethylene-responsive factor) superfamily. DREBs respond to dehydration stress through functioning in the modulation of both ABA-independent and ABA-dependent pathways. DREB1A interacts with ABF4, resulting in the regulation of an ABA-independent pathway [40]. The overexpression of *OsDREB1F* from rice confers tolerance to salt, drought, and low temperature in Arabidopsis and rice as a consequence of the activation of ABA-dependent stress-inducing genes, such as *RD29B* and *RAB18* [47]. In the present study, *DREB1A* was suppressed prior to 4 h but was highly induced at 8 h, 12 h and 24 h (Fig 8i). Therefore, we hypothesize that *DREB1A* may be indirectly regulated by *SnRK2*.*2* in the early stage or may interact with *ABF4* in the middle phase.

Additionally, SnRK2.2 shows a close relationship with SnRK2.6 in Arabidopsis [48]. Moreover, SnRK2.6 can increase drought tolerance by phosphorylating an inward K^+^ channel 3-ketoacyl-CoA thiolase-1 (KAT1) [49]. The overexpression of *TaSnRK2*.*3* from wheat (*Triticum aestivum* L.) in transgenic *Arabidopsis* increases tolerance to drought and salt stresses via the regulation of ABA stress-related genes [50]. Similarly, the overexpression of *GhSnRK2* from *Gossypium hirsutum* in transgenic Arabidopsis improves drought and low-temperature tolerance through an ABA stress-responsive pathway [51]. In the presence of PEG, the expression of *SnRK2*.*2* in W1-1, W1-2 and wild-type plants was found to be significantly inhibited at 1 h, 8 h and 36 h and markedly induced at 12 h and 24 h. In addition, its expression in the two transgenic lines was markedly higher than that observed in the wild-type plants (Fig 8d). In agreement with previous evidence regarding the variation in *PP2C*, the observed increase in the repression of *SnRK2*.*2* corresponded to increasing levels of *PP2C*. Furthermore, *CmWRKY1* may synergistically interact with *SnRK2*.*2* to a certain extent. As ABA binds to PYL2, the activation of *PP2C*, *ABI1*, and *ABI2* can be suppressed, and as a result, the activity of *SnRK2*.*2* can be facilitated [52]. *SnRK2*.*2* has been reported to be involved in the phosphorylation and activation of *ABF4* [40]. We found that *CmWRKY1* inhibits *PP2C*, *ABI1*, and *ABI2*, alleviating the effect on *SnRK2*.*2* and thereby contributing to dehydration tolerance.

### Stimulation of ABA-Associated Genes, Such as *NCED3A*, *PYL2*, *RAB18*, and *DREB1A*, by *CmWRKY1*

Plants respond to dehydration stress through both ABA-independent and ABA-dependent pathways. DREB1A responds to dehydration stress through ABA-dependent pathways [40]. In the present study, *DREB1A* was found to be highly induced by *CmWRKY1*. The crosstalk of the ABA-dependent pathway is markedly more complicated. *NCED3A* is rapidly induced to synthesize endogenous ABA following drought stress [53]. In this study, *NCED3A* from chrysanthemum was shown to be significantly induced by *CmWRKY1*. *RAB18* and *MYB2* have been recognized as important players with positive roles in the ABA signaling network [14].

*NCED3*, short for 9-cis-epoxycarotenoid dioxygenase 3, is rapidly induced to synthesize endogenous ABA following drought stress. A previous investigation demonstrated that the overexpression of *AtNCED3* in transgenic Arabidopsis leads to an enhancement of the endogenous ABA level and the induction of ABA stress-responsive genes [8]. A recent study suggested that *AtNCED3* is markedly activated by the water deficit status in Arabidopsis [53]. In the present study, *NCED3A* from chrysanthemum was observed to be significantly induced in both transgenic and wild-type chrysanthemum in the presence of PEG. The expression of *NCED3A* was found to be markedly greater in transgenic chrysanthemum than in wild-type plants at 4 h, 8 h and 12 h (Fig 8a). Based on the obtained evidence, it is obvious that dehydration strongly induces *NCED3A* to biosynthesize ABA in response to a series of physiological and cellular processes in order to combat water deficit stress. In this study, we observed that *CmWRKY1* may stimulate *NCED3A* in the early stage of water loss. As ABA increased to a certain level, the expression of *NCED3A* in the transgenic lines and wild-type plants decreased, indicating that the increase in endogenous ABA may have a negative effect on the expression of *NCED3A* and *CmWRKY1*.

The expression of *PP2C* was markedly higher in both the overexpression lines and the wild-type plants under dehydration. In addition, the transcript levels in W1-1 and W1-2 were markedly lower than those observed in the wild-type plants before the 12-h treatment point, but after this time point, the level in the transgenic plants was higher (Fig 8c). These findings are different from those obtained for *NCED3A* (Fig 8a). Based on our observations, we can hypothesize that *PP2C* is strongly reduced by dehydration treatment. *CmWRKY1* may function in the ABA-signaling pathway in response to PEG stress by mediating the expression of either *NCED3A* or protein phosphatase 2C (PP2C).

ABA interacts with pyrabactin resistance 1-like (PYL) and other pyrabactin resistance (PYR)-related receptors as well as the regulatory component of the ABA receptor (RCAR), which often work together as a structure in the presence of ABA. As ABA binds to PYL/PYR/RCAR, the activation of *PP2C* can be suppressed, and as a result, the activity of subclass III sucrose non-fermenting-1 (SNF1)-related protein kinases 2 (SnRK2s) can be facilitated, which relieves the repression of ABA stress-responsive genes downstream of SnRK2s [52]. It has been widely reported that the composition of ABA-PYL-PP2C-SnRK2s has a predominant effect on their involvement in plant drought tolerance [52,54]. PYL is a member of the START domain family, the members of which have been recognized as pivotal ABA receptors regulating ABA-mediated pathways [55]. For example, PYL5/RCAR3 can increase drought resistance in Arabidopsis through reducing the impact of PP2Cs [56]. Similarly, it has been shown that PYL2 can mediate a sulfonamide ABA agonist referred to as quinabactin, which forms a hydrogen bond with PYL2/PP2C to contribute to interference with stomata opening, reduction of water loss, and improvement of drought tolerance in soybean plants and adult *Arabidopsis* [57]. Our data show that *PLY2* is strongly induced in the transgenic lines compared with the wild-type plants following exposure to PEG (Fig 8b). This result allows us to infer that *CmWRKY1* is likely implicated in plant defense mechanisms against dehydration via the positive regulation of *PLY2*.

*RAB18*, a typical ABA-dependent stress-responsive gene, shows an increase under ABA-associated stress [58]. In this study, the transcript levels of *ABF4/RAB18* were found to be slightly increased prior to the 8-h time point and were highly induced at the 12-h and 24-h time points, in accordance with the data showing that *SnRK2*.*2* was inactivated prior to 8 h and activated thereafter. Moreover, *SnRK2*.*2/ABF4/RAB18* was found to be stimulated to a significantly greater extent in the W1-1 and W1-2 plants than in the wild-type plants following PEG treatment (Fig 8d–8f). The evidence suggests that *CmWRKY1* upregulates ABA-activated regulator genes by modulating a series of connected networks.

*ABI4* (abscisic acid-insensitive 4) belongs to the AP2/ERF superfamily. It has been shown that the overexpression of *ABI4* in Arabidopsis facilitates the susceptibility of plants to high salt levels [59]. Fig 8j demonstrates that *ABI4* was highly induced in the wild-type plants and strongly repressed in the W1-1 and W1-2 plants, but the direct link between *CmWRKY1*and *ABI4* remains unknown.

Based on the evidence presented above, *CmWRKY1* likely enhances dehydration tolerance by regulating the interaction of the ABA-PYL-PP2C-SnRK2 complex. Under dehydration stress, many ABA-responsive genes, such as *ABF4*, *ABI5*, *RAB18*, and *MYB2*, are induced. In this study, the transcript levels of *ABF4*, *ABI5*, *RAB18*, and *MYB2* in W1-1 and W1-2 plants were found to be distinctly altered to varying degrees compared with the levels obtained in the wild-type plants following exposure to PEG (Fig 9).

In this study, the mechanism investigated provides a clue for studying the crosstalk between *CmWRKY1* and ABA involved in dehydration stress in chrysanthemum. However, the underlying mechanisms that fine-tune the direct interaction between *CmWRKY1* and ABA-associated genes to regulate water loss stress remain to be elucidated. Future research on the basic mechanisms involved in this complicated cross-talk will increase our knowledge of the interactions between *CmWRKY1* and ABA stress-responsive genes involved in the dehydration tolerance of chrysanthemum.

## Conclusions

CmWRKY1, a group IIb WRKY family member isolated from *Chrysanthemum morifolium*, exhibits no transcriptional activation in yeast cells. The examination of its subcellular localization showed that CmWRKY1 is localized to the nucleus *in vivo*. Furthermore, *CmWRKY1*-overexpressing transgenic lines were found to exhibit enhanced dehydration tolerance under polyethylene glycol (PEG) treatment compared with wild-type plants. We further confirmed that the transgenic plants presented suppressed expression of genes negatively regulated by ABA, such as *PP2C*, *ABI1* and *ABI2*, and activated expression of genes positively regulated by ABA, such as *PYL2*, *SnRK2*.*2*, *ABF4*, *MYB2*, *RAB18*, and *DREB1A*. Our results indicate that *CmWRKY1* plays an important role in the response to drought in chrysanthemum through an ABA-mediated pathway.

## Supporting Information

S1 Table Names and sequences of the primers used in this study. (PDF)
