# Supplementary material for: CmWRKY1 Enhances the Dehydration Tolerance of Chrysanthemum through the Regulation of ABA-Associated Genes
Source: PLoS One. 2016 Mar 3;11(3):e0150572. doi: 10.1371/journal.pone.0150572 (PMC4777562; doi:10.1371/journal.pone.0150572)
Supplement: S1 Table — (PDF) [file pone.0150572.s001.pdf]

Table 1 Primer names and sequences used in this study.

Prime name	Sequence (5′ to 3′)
CmHyg-F	CTTCTACACAGCCATCGGTCCAG
CmHyg-R	CGGAAGTGCTTGACATTGGGGAG
Oligo (dT)	AAGCAGTGGTATCAACGCAGAGTACTTTTTTTTTTTTTTTT
dT-R	AAGCAGTGGTATCAACGCAGAGTAC
CmEF1α-F	TTTTGGTATCTGGTCCTGGAG
CmEF1α-R	CCATTCAAGCGACAGACTCA
CmABI4-F	GATCGTGGCTGAGAGACTCG
CmABI4-R	TACCCCACGTTCTTTGCCTC
CmABI5-F	CAGCAGCAGCAACAACTGTT
CmABI5-R	CTACAGGACTACCCAACGGC
CmDREB1A-F	CGGTTTTGGCTATGAGGGGT
CmDREB1A-R	TTCTTCTGCCAGCGTCACAT
CmMYB2-F	GGCCATGGACACTTGAGGAA
CmMYB2-R	ATTCCAACGGCCTTCACCAT
CmRAB18-F	TGTAGTGCAAAAACCCGGGT
CmRAB18-R	GTTGACCCCTCAGCAGTCAA
CmABI1-F	ATGGGCATCGTGTTTTTGGC
CmABI1-R	TAACCCGTCACTGGCTAGGA
CmPYL2-F	TCAATCCCACAAGGCCTCAC
CmPYL2-R	ACGAGGGATGTGCATGTGTT
CmABI2-F	GAAGAGCCCGGGTCTGAAAA
CmABI2-R	CGCCAATGCCAACTTTGTCA
CmSnRK2.2-F	ATTGTTGGATGGGAGCCCTG
CmSnRK2.2-R	GCTGGTGTACCAACTGTCGA
CmPP2C-F	TAGTGATGGACTTTGGGATGTGG
CmPP2C-R	GCCAATGCCAACTTTGTCAGTAA
CmNCED3A-RT-F	AGTATGGTGGTGAGCCGTTGTATCTAC
CmNCED3A-RT-R	GCATTCACAATCTGGAGTTCGGACTTC
CmWRKY1-GATE-SAL-F	CGCGTCGACATGGGCAAAGATGGA
CmWRKY1-GATE-NOT-R	TTTGCGGCCGCGAATTTCCTTGGAAAT
CmWRKY1-DL-F	CCACGTTTGAATTCTTCGGT
CmWRKY1-DL-R	TCGCGATGTGAGTTCTTGTC
